# Intermediate Monocytes and Cytokine Production Associated With Severe Forms of Chagas Disease

**DOI:** 10.3389/fimmu.2019.01671

**Published:** 2019-07-19

**Authors:** Sergio Gómez-Olarte, Natalia I. Bolaños, Mariana Echeverry, Ayda N. Rodríguez, Adriana Cuéllar, Concepción J. Puerta, Alejandro Mariño, John M. González

**Affiliations:** ^1^Grupo de Ciencias Básicas Médicas, School of Medicine, Universidad de los Andes, Bogotá, Colombia; ^2^Department of Biological Sciences, School of Sciences, Universidad de los Andes, Bogotá, Colombia; ^3^National Blood Bank, Colombian Red Cross, Bogotá, Colombia; ^4^Grupo de Ciencias del Laboratorio Clínico, School of Sciences, Pontificia Universidad Javeriana, Bogotá, Colombia; ^5^Laboratorio de Parasitología Molecular, School of Sciences, Pontificia Universidad Javeriana, Bogotá, Colombia; ^6^Failure and Heart Transplantation Clinic, Hospital Universitario San Ignacio, Bogotá, Colombia

**Keywords:** cardiomyopathy, Chagas disease, chemokines, cytokines, monocytes, innate immunity

## Abstract

Monocytes are classified according to their CD14 and CD16 expression into classical (reparative), intermediate (inflammatory), and non-classical. This study assessed the frequency of monocyte and the relationship between monocyte subset percentages and the levels of blood cytokines in Colombian chagasic patients with different clinical forms. This study included chagasic patients in different clinical stages: indeterminate (IND) *n* = 14, chronic chagasic cardiomyopathy (CCC) *n* = 14, and heart transplant chagasic (HTCC) *n* = 9; controls with non-chagasic cardiopathy (NCC) *n* = 15, and healthy individuals (HI) *n* = 15. Peripheral blood mononuclear cells (PBMCs) were isolated, labeled for CD14, CD16, and HLA-DR, and analyzed by flow cytometry. Cytokines were measured with a bead-based immunoassay. Percentages of total CD14+ CD16+ and CD14+ HLA-DR+ monocytes were higher in patients with heart involvement (CCC, HTCC, and NCC) than controls. Percentages of intermediate monocytes increased in symptomatic chagasic patients (CCC and HTCC) compared to asymptomatic chagasic patients (IND) and controls (HI). Asymptomatic chagasic patients (IND) had higher percentages of classical monocytes, an increased production of CCL17 chemokine compared to chagasic symptomatic patients (CCC), and their levels of CCL17 was positively correlated with the percentage of classical monocyte subset. In CCC, the percentages of intermediate and classical monocytes were positively correlated with IL-6 levels, which were higher in this group compared to HI, and negatively with IL-12p40 concentration, respectively. Remarkably, there also was an important increased of classical monocytes frequency in three chronic chagasic patients who underwent cardiac transplant, of which one received anti-parasitic treatment. Our findings suggest that cardiac chagasic patients have an increased percentage of inflammatory monocytes and produce more IL-6, a biomarker of heart failure and left ventricular dysfunction, whereas asymptomatic chagasic individuals present a higher percentage of reparative monocytes and CCL17.

## Introduction

Chagas disease (CD) is a public health problem in Latin-America with an estimated prevalence of 10 million cases in 2015 (1), and the major cause of infectious cardiomyopathy worldwide (2). Even though 70% of infected individuals remain asymptomatic during their lifetime (3), 20–30% will develop the chronic chagasic cardiomyopathy (CCC), an inflammatory event in which *Trypanosoma cruzi*, the parasitic agent, persists in the heart leading to multifocal inflammation, ischemia, and necrosis. The cardiac tissue alterations progress to congestive heart failure, a condition characterized by dilatation and hypertrophy of the heart ventricles associated with a high mortality rate (4). Among the myocardial-infiltrating cells, there are T cells and antigen presenting cells (APCs); CD8+ T cells are the most abundant cells followed by CD4+ T cells and macrophages, favoring heart inflammation, tissue remodeling, and cellular damage (5). Although the role of CD8+ T cells is protective during the acute phase (6, 7), they fail to control *T. cruzi* during chronic infection (6). T cells from peripheral blood or heart tissue of chagasic patients display an exhaustion phenotype, characterized by alterations in their proliferation capacity, increased expression of inhibitory markers and progressive loss of cytokine secretion (8–13), which are associated with parasite persistence (8, 14, 15). Although less abundant, APCs such as dendritic cells and monocytes/macrophages, can interact with parasite antigens modifying their antigen presentation and their cytokine profile secretion (16–25).

Monocytes are heterogeneous and multifunctional cells that convey great plasticity; as innate immune cells, they phagocyte microbes and produce oxygen reactive species (26, 27), and also participate in cellular processes including tissue repair and regeneration during heart diseases (4, 28). The increase of peripheral blood monocytes has been associated with left ventricular remodeling after acute myocardial infarction (29). Cytokines produced by monocytes including TNF-α and IL-6 and soluble CD14 are correlated with severe congestive heart failure (30–32); also soluble TNF receptor can be a predictor of mortality in the same condition (33, 34). Inflammatory monocytes (CD16+) have been associated with the pathogenesis of several infectious diseases including some caused by parasites (35–40). Currently, monocytes are phenotypically classified into three subsets according to the CD14 (bacterial LPS receptor) and CD16 (IgG low affinity receptor) expression: classical (CD14++ CD16–), intermediate (CD14++ CD16+), and non-classical monocytes (CD14+ CD16++) (41, 42). Each of these subsets can have distinct functions. Intermediate monocytes participate in angiogenesis and have a high expression of MHC class II. Classical monocytes specialize in IL-10 production and phagocytosis (35), and are involved in tissue repair. Non-classical monocytes are characterized by their patrolling activity, and both non-classical and intermediate subsets are the main sources of pro-inflammatory cytokines such as TNF-α, IL-1β, IL-6, and IL-8 (35, 43, 44). Several studies support the pivotal role of monocytes-mediated inflammation in the pathogenesis of cardiomyopathy and the progression of congestive heart failure (30).

*T. cruzi* induces phenotypic and functional alterations in monocytes during the acute and chronic phases of CD. During the acute infection, monocytes present an augmented expression of HLA-DR, TLR2, and costimulatory CD80/CD86 molecules, and increase the production of TNF-α, IL-12/IL-23p40, and IL-10 (45). In chronic stages of the disease, there has been found a correlation between MHC class II, co-stimulatory molecules expression and cytokines production with the development of CCC (16, 17, 19–25).

Only few studies in Chagas disease have characterized the phenotype of the monocyte subsets based on CD14 and the CD16 expression, finding an increase of CD14+ CD16+ monocytes percentage in asymptomatic children (46, 47) and adults during acute and chronic infection (48–50). In this work, we studied the variations in the frequency of the total monocyte population, and the correlation between monocyte subsets and the seric levels of IL-1β, IL-1RA, IL-6, IL-10, IL-12p40, IL-12p70, IL-23, TNF-α, CCL17, and CXCL10 in samples from chronic chagasic patients with different stages of the disease.

## Materials and Methods

### Ethics Statement

Research protocol and informed consent were approved by the Ethical Committees of Universidad de los Andes (458-2015) and Hospital Universitario San Ignacio (06-2016) from Bogotá, Colombia. The protocol follows the Colombian national regulations and the Declaration of Helsinki. All the included subjects provided written informed consent.

### Human Donors

Thirty-eight cardiomyopathy patients were recruited at the Failure and Heart Transplantation Clinic in the Hospital Universitario San Ignacio, of which 23 had chronic Chagas disease. Chagasic patients were classified in two groups, IND or asymptomatic chagasic (stages A and B) and CCC or chronic chagasic cardiomyopathy (stages C and D) according to the American College of Cardiology/American Heart Association staging. IND group included 14 individuals, 6 males and 8 females with ages ranging from 28 to 62 years. CCC group included 14 patients, 7 males and 7 females with ages ranging from 53 to 78 years. A third group of 9 heart transplant chagasic patients (HTCC), 5 males and 4 females with ages ranging from 50 to 71 years, were patients who had received a heart transplant from 3 weeks up to 13 years before entering the study. The control groups included 15 patients with non-chagasic myocardiopathy (NCC), 9 males and 6 females with ages from 42 to 72 years, and 15 healthy individuals (HI), 7 males and 8 females with ages from 34 to 62 years old and seronegative for *T. cruzi* antibodies. All volunteers were tested for antibodies against *T. cruzi* by immunofluorescence indirect assay (IFI) with trypomastigotes and an ELISA kit for IgG anti-*T. cruzi* antibodies (NovaTec, Dietzenbach, Germany) (51). The exclusion criteria were chronic inflammatory conditions or acute infections during the time of sampling. Characteristic of individuals are shown in Table 1.

**Table 1:** Demographic and clinical characteristics of patients and individuals enrolled in the study.

	**IND**	**CCC**	**HTCC**	**NCC**	**HI**	***p-*value**
Number of individuals	14	14	9	15	15	—
Age mean (±SD)	49.7 (9.8)	63.6 (8.8)	60.8 (8.5)	63.9 (10.3)	53.6 (8.2)	0.0013^*^
Sex male %	43	50	56	60	47	—
ACC/AHA classification						
A No.	12	—	—	—	—	—
B No.	2	—	—	—	—	—
C No.	—	11	4	15	—	—
D No.	—	3	5	—	—	—
Mean LVEF % (±SD)	58.8 (6.3)	27.2 (11.5)	30.0 (22.8)	27.3 (11.1)	—	0.0340^**^
Heart failure etiology						
Ischemic heart failure	—	—	—	10	—	—
Hypertensive heart failure	—	—	—	2	—	—
Idiopathic heart failure	—	—	—	2	—	—
Congenital heart failure	—	—	—	1	—	—

*Groups. Asymptomatic or indeterminate chagasic patients (IND): those with non-structural cardiac damage (groups A and B); symptomatic or cardiomyopathy chagasic patients (CCC): those with structural cardiac damage (groups C and D); heart transplant chagasic patients (HTCC): chagasic patients who received a new heart in the last 13 years; non-chagasic myocardiopathy (NCC): patients with congestive heart failure associated with an etiology other than CD; healthy individuals (HI). American College of Cardiology/American Heart Association (ACC/AHA) classification. A: normal electrocardiogram (ECG) and echocardiogram (ECHO) findings, and New York Heart Association (NYHA) functional classification I; B: abnormal ECG findings, normal ECHO and NYHA I; C: abnormal ECG findings, increased heart size, decreased left ventricular ejection fraction (LVEF) and NYHA II or III; D: same as class C, but NYHA IV*.

* *The IND group had a lower age than CCC (p = 0.0107) and NCC (p = 0.0068) patients*.

** *The IND had higher LVEF compared to CCC (p = 0.0387) and HTCC (p = 0.0417) groups*.

### Blood Sample, Isolation of Peripheral Blood Mononuclear Cells and Cell Surface Staining

Blood samples were drawn in EDTA vacutainer tubes (BD Biosciences, Franklin Lakes, NJ, USA). Peripheral blood mononuclear cells (PBMCs) were isolated by density gradient centrifugation with Ficoll-Hypaque (GE Healthcare, Chicago, IL, USA) and their viability was evaluated with trypan blue 0.4% (Cell Signaling Technology, Danvers, MA, USA). One million of PBMCs were incubated with anti-CD16/32 (clone 2.4G2) from Tonbo Biosciences (San Diego, CA, USA) and human AB serum to block Fc receptors, and then stained with anti-CD14 PE (HCD14) and anti-CD16 PE-Cy7 (3G8) purchased from BioLegend (San Diego, CA, USA) and anti-HLA-DR FITC (Tu39) from BD Biosciences or with their corresponding isotype controls. Stained samples were incubated for 25 min at 4°C and acquired in a FACS Canto II cytometer with FACSDiva software (BD Biosciences). At least 50,000 events were acquired on the HLA-DR+ gate; over this population it was defined the percentage of monocytes (CD14+ CD16+) and the monocyte subsets according to the CD14 and CD16 expression as follows: CD14++ CD16- (classical), CD14++ CD16+ (intermediate), and CD14+ CD16++ (non-classical) (see Figure 2A). Percentages of HLA-DR+ and HLA-DR mean fluorescence intensity (MFI) were measured in each monocyte subset. Gating strategy is shown in Supplementary Figure 1. Flow cytometry files for monocytes staining can be found in the FlowRepository (https://flowrepository.org/id/FR-FCM-ZYTR).

### Detection of Cytokines in Human Plasma

The quantification (pg/mL) of proinflammatory (IL-1β, IL-6, IL-12p40, IL-12p70, IL-23, and TNF-α) and regulatory (IL-1RA and IL-10) cytokines and chemokines (CXCL10 and CCL17) in the plasma of all donors were carried out with LEGENDplex™ Human M1/M2 Macrophage Panel (10-plex) from BioLegend. The assay was done according to the manufacturer's instructions. The samples were acquired on a FACS Canto II flow cytometer and the data was analyzed with the LEGENDplex™ v8.0 software.

### Statistical Analysis

Descriptive statistics were used to depict the population and to present the flow cytometry data. Data distribution was evaluated by the Shapiro-Wilk test. Non-parametric analyses were used to compare multiple groups using the Kruskal Wallis test followed by Dunn's *post-hoc* test. The Mann-Whitney test was used for non-parametric comparison of the cytokines levels. The correlation between the percentage of monocyte subsets of each group and the cytokines concentration were evaluated by the Spearman's coefficient. All statistical analysis was carried out with the GraphPad Prism 7.0 software (GraphPad, San Diego, CA, USA) considering *p*-values < 0.05 as significant.

## Results

### Patients Characteristics

Asymptomatic individuals (IND) were younger than patients with heart involvement (*p* = 0.0013), but have a similar age to the healthy individuals (HI). Left ventricular ejection fraction (LVEF) had normal ranges in IND individuals. All patients with cardiomyopathy (CCC, HTCC, and NCC) had an LVEF <50%. The individuals in the NCC group allows to determine if changes could be related to *T. cruzi* infection or the heart disease itself (Table 1). The median OD_450_ of anti-*T. cruzi* antibodies by ELISA in chagasic patients was similar between CCC, IND, and HTCC groups (OD_450_ values of 2.857, 2.844, and 2.799, respectively), whereas the controls presented a median OD_450_ below the cut-off (0.267 and 0.210 for NCC and HI) as expected. All chagasic patients were reactive by indirect immunofluorescence (IFI) with trypomastigotes at 1:40 dilution.

### Chagasic Patients With Heart Disease Displayed Higher Percentages of Total CD14+ CD16+ and CD14+ HLA-DR+ Monocytes

**Figure 1:**
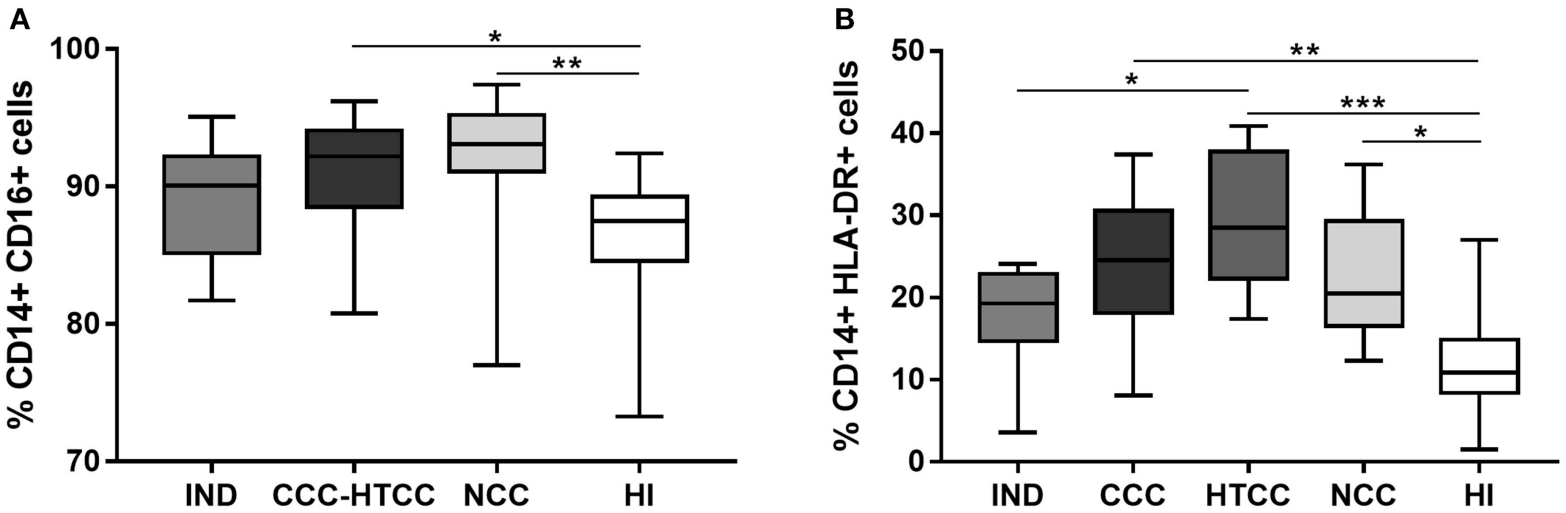
Analysis of CD14+ CD16+ **(A)** and CD14+ HLA-DR+ **(B)** cells percentages from peripheral blood of chagasic patients classified as IND, CCC, and HTCC, and controls defined as NCC and HI. Multiple comparisons were done with the Kruskal-Wallis test followed by Dunn's *post-hoc* test. Differences are indicating with: ^*^p < 0.05, ^**^p < 0.01, ^***^p < 0.001.

It was assessed the percentages of monocytes and their HLA-DR expression in peripheral blood cells from all donors. The total monocytes percentage, defined as CD14+ and CD16+ cells, increased in donors with heart involvement: CCC-HTCC (*p* = 0.00164) and NCC patients (*p* = 0.0034) compared to HI group (Figure 1A). With the aim to compare with previous studies, it was analyzed the percentages of CD14+ HLA-DR+ cells, which were also higher in patients with heart damage, including CCC (*p* = 0.0030), HTCC (*p* < 0.0001), and NCC (*p* = 0.0129), compared to HI group (Figure 1B). When the total percentages of CD14+ CD16+ cells in the CCC (*p* = 0.1352) and HTCC (*p* = 0.0765) groups were analyzed separately, there was no significant difference compared to HI.

### Intermediate Monocytes Increased in Cardiac Chagasic Patients

**Figure 2:**
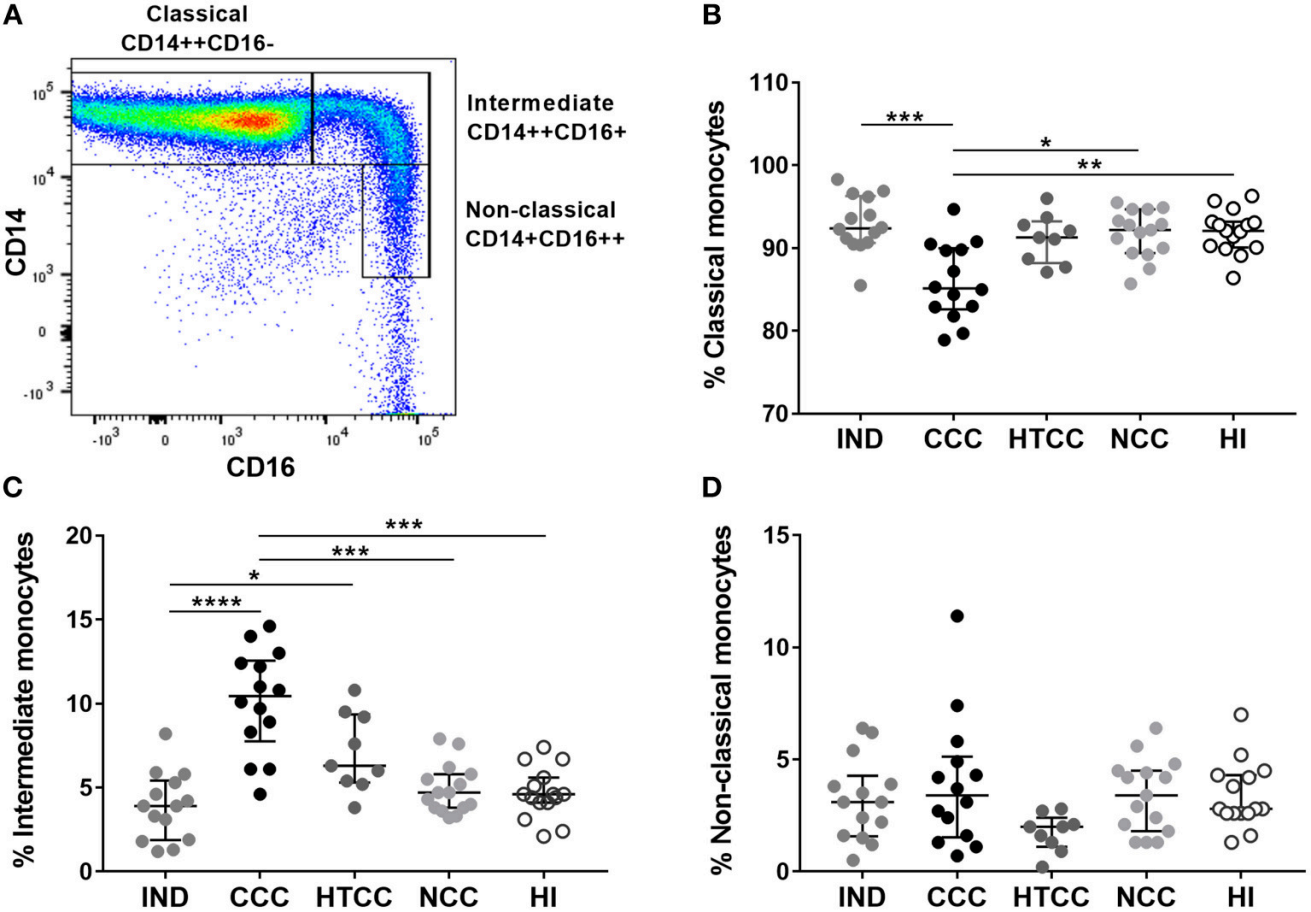
Analysis of normalized percentages of monocyte subsets in IND (*n* = 14), CCC (*n* = 14), and HTCC (*n* = 9) patients and NCC (*n* = 15) and HI (*n* = 15) controls. The strategy used to differentiate the three monocyte subsets according to CD14 and CD16 expression is shown in **(A)**. The percentages of classical **(B)**, intermediate **(C)**, and non-classical **(D)** monocytes between the groups were compared with the Kruskal-Wallis test followed by Dunn's *post-hoc* test. The values are presented by median and interquartile ranges and differences are indicating with: ^*^p < 0.05, ^**^p < 0.01, ^***^p < 0.001, ^****^p < 0.0001.

As monocytes increased in chagasic and non-chagasic individuals with heart disease, we determined the frequency of classical, intermediate, and non-classical monocytes according to CD14 and CD16 expression (Figure 2A). It was found that CCC patients had a reduced percentage of classical monocytes compared to IND (*p* = 0.0007), NCC (*p* = 0.0121), and HI (*p* = 0.0074) groups, but without differences with HTCC (Figure 2B). There was an increase of intermediate monocytes percentage in CCC patients compared to IND (*p* < 0.0001), NCC (*p* = 0.0009), and HI (*p* = 0.0004). Also, there was an augmented frequency of intermediate monocytes in HTCC patients only compared to IND individuals (*p* = 0.0375) (Figure 2C). Non-classical monocytes had similar percentages (*p* > 0.05) in all groups (Figure 2D). HLA-DR intensity (MFI) of classical, intermediate and non-classical monocytes was similar (*p* > 0.05) among groups, however, classical monocytes in the IND group had a higher HLA-DR MFI compared to HI (*p* = 0.0054). Additionally, MFI values for HLA-DR in each group was higher in intermediate monocytes as expect, since this monocyte subset expresses more MHC class II molecules (Supplementary Figure 2).

### IL-12p40 and CCL17 Production Decreased in Cardiac Chagasic Patients

**Figure 3:**
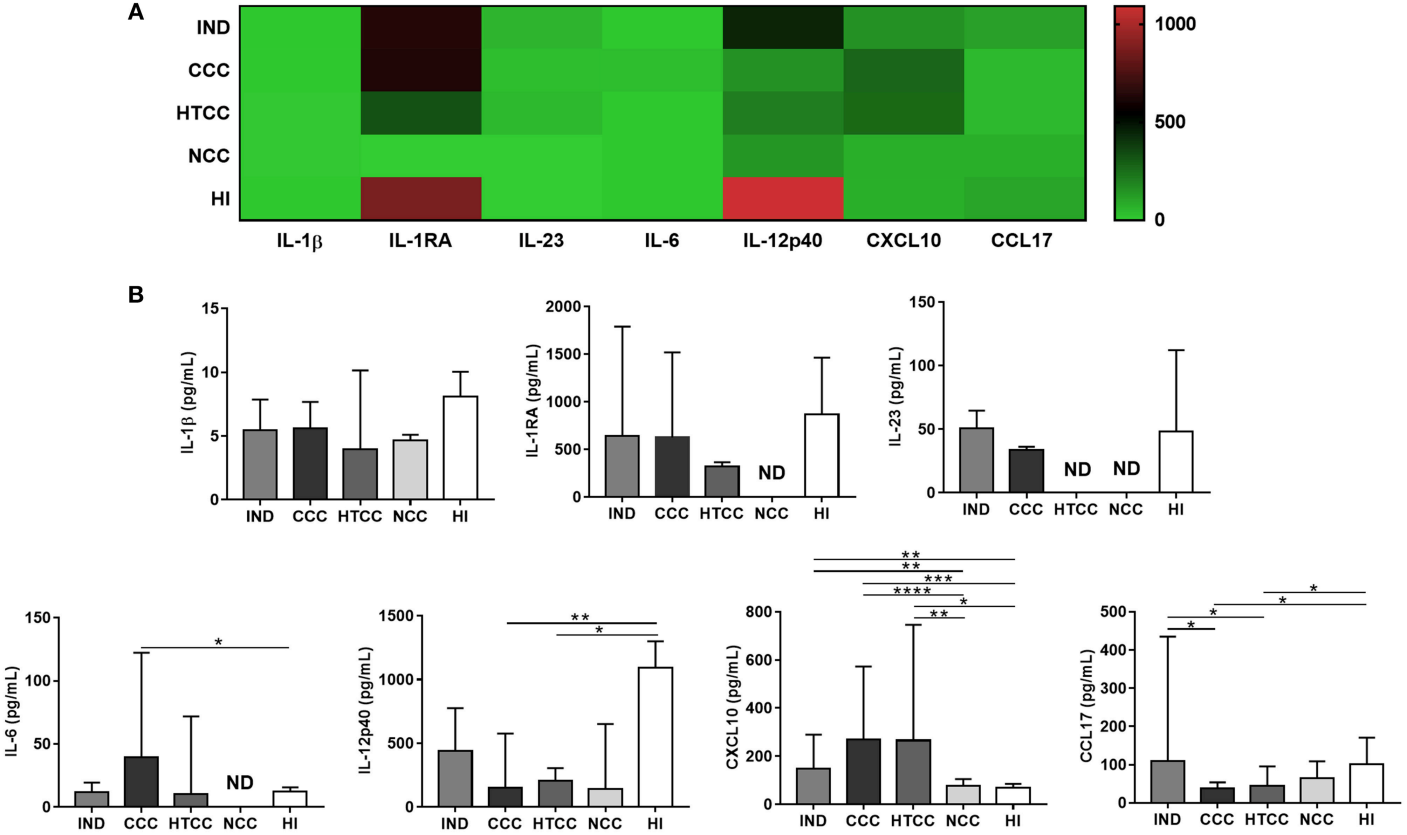
Cytokines and chemokines concentration in plasma from donors of all groups. Heat map that shows the patterns of cytokines and chemokines concentration in chagasic patients (IND, CCC, and HTCC) and controls (NCC and HI) are distinct **(A)**. Comparison of IL-1β, IL-1RA, IL-6, IL-12p40, IL-23, CXCL10, and CCL17 levels was done with Mann-Whitney test. The median and interquartile ranges of cytokines are displayed **(B)**. ND, Not detectable. Differences are indicated as follows: ^*^p < 0.05, ^**^p < 0.01, ^***^p < 0.001, ^****^p < 0.0001.

To evaluate if inflammatory and regulatory cytokines and chemokines were differentially secreted, the levels of IL-1β, IL-1RA, IL-6, IL-10, IL-12p40, IL-12p70, IL-23, TNF-α, CCL17, and CXCL10 were measured in plasma. The patterns of cytokine and chemokine production distinguished chagasic patients and controls; for example, HI tend to have more IL-12p40 and IL-1RA, meanwhile chagasic patients (IND, CCC, and HTCC) had higher concentrations of chemokines (CCL17 and CXCL10) (Figure 3A). TNF-α was only detectable in control groups: NCC (mean of 12.73 pg/mL) and HI (mean of 12.45 pg/mL), whereas the concentration of this cytokine in chagasic patients was below the detection limit (3 pg/mL). Similarly, IL-10 and IL-12p70 concentration in all donors were below the detection limit (4.52 and 4.26 pg/mL, respectively), except for two IND donors with a mean IL-12p70 value of 19.55 pg/mL. IL-6 was the only cytokine that showed increased levels in CCC compared to HI (*p* = 0.0480). In contrast, IL-12p40 levels in CCC and HTCC patients was reduced compared to HI controls (*p* = 0.0052 and *p* = 0.0336). Regarding chemokines, CXCL10 concentration was higher in chagasic patients (IND, CCC, and HTCC groups) compared to NCC (*p*-values of 0.0031, <0.0001, and 0.0055, respectively) and HI (*p*-values of 0.0083, 0.0001, and 0.0148, respectively). CCL17 had decreased levels in CCC and HTCC patients compared to IND (*p* = 0.0160 and 0.0111) and HI (*p* = 0.0246 and 0.0349), respectively (Figure 3B).

### Intermediate and Classical Monocytes Correlated With IL-6 and CCL17 Production in Chagasic Patients

**Figure 4:**
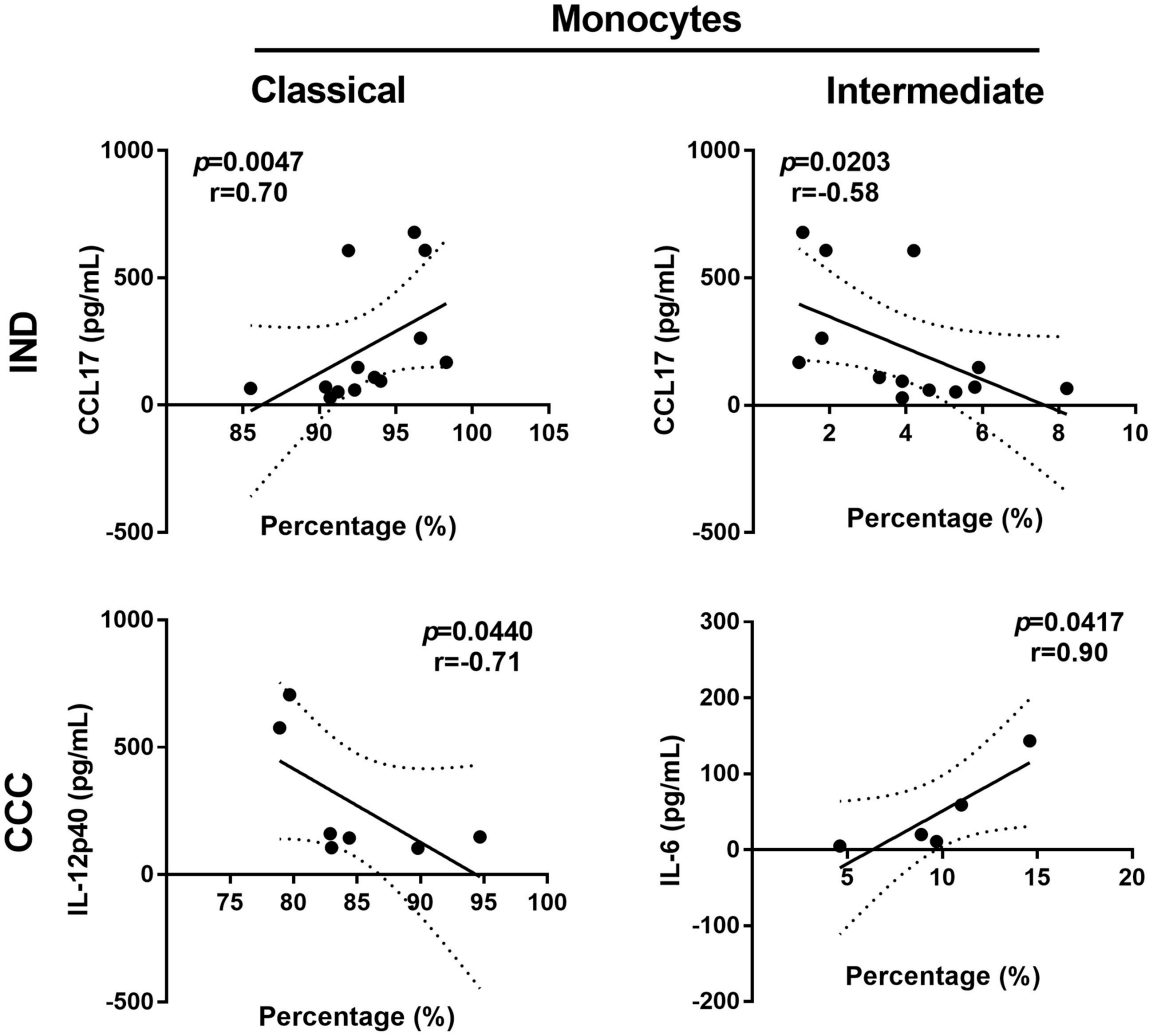
Scatter plot showing the followings correlations: percentages of classical monocytes and CCL17 concentration in IND, percentages of intermediate monocytes and CCL17 concentration in IND, percentages of intermediate monocytes and IL-6 concentration in CCC. Spearman's rank correlation coefficient (*r*) and *p-*values were calculated for every association using non-parametric Spearman's correlation test. IND, indeterminate; CCC, chronic chagasic cardiomyopathy.

It was evaluated the correlation between monocyte subset percentages and cytokines concentration in all groups. In CCC, it was observed a positive correlation of IL-6 levels and intermediate monocytes (*r* = 1.00; *p* = 0.0167), cytokine and monocyte subset that increased in chronic cardiac patients. In IND donors, augmented CCL17 production had a negative correlation with intermediate monocytes (*r* = −0.58; *p* = 0.0203) and a positive correlation with classical monocytes (*r* = 0.70; *p* = 0.0047) (Figure 4).

### Percentage of Intermediate Monocytes Decreased After Heart Transplant

Two chagasic patients, CCC16 and CCC18, presented a reduction of intermediate monocytes percentages 6 months and 3 weeks, respectively, after receiving the heart transplant; and it was accompanied with augmented IL-12p40 level in plasma (<97.66 pg/mL and 104.30 pre-transplant vs. 329.26 vs. 234.14 post-transplant, respectively). Similarly, the HTCC21 patient with a 10 years transplant, treated twice for *T. cruzi* reactivation with anti-parasitic drugs, showed a lower percentage of intermediate monocytes (5.4%) compared to the mean frequency values of HTCC group (7.1%) as shown in Supplementary Figure 3.

## Discussion

Monocytes/macrophages which are part of the innate immune response are also implicated in tissue inflammation, repair and fibrosis. Indeed, a single population of monocytes can present pro-inflammatory and pro-repair properties (28). Thus, it is important to define the monocyte subsets in parasitic infectious diseases with an inflammatory component. For instance, the intermediate and the classical monocytes are involved in the pathogenesis of both protozoa and helminth diseases, such as leishmaniasis, malaria, schistosomiasis, and filariasis (36–40). The goal of this study was to determine the monocyte subset percentages in peripheral blood and plasma cytokines levels in chagasic patients with different clinical stages.

In this study, CD14+ CD16+ monocytes were higher in chagasic patients with severe disease and in those with heart transplant. A previous study using CD14 as a unique cellular marker did not show any difference in cells frequencies from chronic chagasic patients (17). As antigen-presenting cells, monocytes process and present *T. cruzi* peptides onto MHC class II molecules in order to activate T cells (3), therefore we assessed CD14+ HLA-DR+ cells frequency, as described by others (17, 47). It was observed an increased percentage of these cells in all cardiac patients, chagasic or not. One study described a reduction of CD14+ HLA-DR+ cells in indeterminate chagasic compared to healthy individuals (17). Here indeterminate individuals had lower percentages of CD14+ HLA-DR+ cells only compared to transplanted chagasic patients. An increase of CD14+ CD16+ HLA-DR+ monocytes and CD14+ HLA-DR+ cells were described in the early chronic phase in children and in chronic chagasic adults after anti-parasitic treatment (46–48). Despite the fact that transplant chagasic patients had different times of grafting and also had immunosuppression, monocyte subsets depicted similar trends among them.

In two previous works, in which the three monocyte subsets were identified in chronic chagasic patients, it also was found an increase of intermediate monocytes in cardiac patients (49, 50); although, we report different results regarding classical and non-classical monocytes percentages. These differences could be attributed to sample processing, staining reagents and distinct gating strategies for defining the subsets (52). Interestingly, intermediate and classical monocytes are associated with worse prognosis in different myocardial diseases. A higher frequency of intermediate monocytes is correlated with lower LVEF in patients with congestive heart failure (53), elevation of ST segment after myocardial infarction and IL-6 production (54). Similarly, the increased percentage of classical monocytes are correlated with LVEF reduction 6 months after acute myocardial infarction (55).

The intermediate monocytes are characterized by a higher expression of MHC class II molecules as shown here, and CD40 co-stimulatory molecule (43). They also secrete inflammatory cytokines such as IL-6, IL-1β, and mainly TNF-α (56), reflecting their role in inflammation and promotion of T cells activation. Some patterns of cytokines and chemokines in peripheral blood were found in this study. Remarkably, IL-12p40 and CCL17 (TARC), molecules related to T cells response (57, 58), had reduced levels in patients with severe form of Chagas disease. Similarly, these patients had augmented intermediate monocytes and increased production of IL-6, a proinflammatory cytokine (59). CXCL10 or interferon gamma-induced protein 10 (IP-10), a chemokine secreted by monocytes in response to IFN-γ (60), is augmented in all chagasic patients. In contrast, asymptomatic individuals presented an increased percentage of reparative monocytes (classical) and a higher level of CCL17. In chronic chagasic patients IL-12 expression is decreased in antigen-stimulated monocytes. Furthermore, *ex vivo* expression of IL-12 was higher on classical monocytes of asymptomatic individuals compared with uninfected controls (49). Macrophage-derived IL-12 is crucial to control parasitemia during acute infection (61), as also shown in an IL-12p40 KO murine infection model (62). A study showed CCL17 mRNA was up-regulated in chagasic myocardium, but its genetic polymorphisms were not associated with risk of cardiomyopathy development (63). According to our data, IL-12 and CCL17 levels were higher in asymptomatic patients compared to cardiac chagasic patients. Indeed, it has been proposed that Th2 cytokines reduced production and high levels of Th1 cytokines are associated with chagasic cardiomyopathy severity (64). As reported, IL-12 is crucial for Th1 populations development, and although Th1 role could be controversial in Chagas disease (64), IFN-γ has been associated with protection (65). Here, cardiac chagasic patients displayed higher seric concentration of IL-6 (66), cytokine associated with pro-fibrotic factors and cardiac deterioration in chagasic children and adults (59, 67). Similarly, CXCL10 plasma levels were higher in both asymptomatic and symptomatic chronic chagasic patients compared to controls (68). This chemokine is also considered a predictor of severity in parasitic infections (60). The CXCL10 mRNA expression increased in hearts from chronic *T. cruzi* infected dogs (69) and humans (63, 70). Interestingly, there is an IFN-γ independent CXCL10 activation pathway, because its gene contains binding sites for multiples pro-inflammatory transcriptions factors (71).

Of note, we had access to samples of two chagasic patients before and after heart transplant. After the transplant and without anti-parasitic treatment, these two patients displayed a reduction of intermediate monocyte percentages (Δ: 3.4 and 5.1%) and IL-12p40 increased production, suggesting the recovery of a protective immune response after heart transplant. Intermediate monocytes frequency changes in adults donors had a lower variation (Δ: 2.3% within 4–6 months), in comparison with the variations in chagasic post-transplant patients showed here (56). Remarkably, one transplant patient who presented reactivation of *T. cruzi* and treated, had a lower value of intermediate monocytes compared to the mean of transplanted patients, indicating that parasite antigen could be responsible for those changes. Due to the low number of samples, this data must be validated in future studies.

In summary, our findings suggest that all cardiac patients, chagasic or not, have augmented percentages of CD14+ CD16+ monocytes, however, the increase of intermediate monocytes were also found in chronic cardiac chagasic patients. Likewise, the associations between the patterns of variation in monocyte subsets and cytokines levels and differential disease stages suggest the potential involvement of these factors either in protection (classical monocytes and IL-12p40/CCL17) or pathogenesis of Chagas cardiomyopathy (intermediate monocytes and IL-6). Functional studies with monocyte subsets during *T. cruzi* chronic infection will be crucial to understand their role in the disease pathogenesis.

## Ethics Statement

Research protocol and informed consent were approved by the Ethical Committees of Universidad de los Andes and Hospital Universitario San Ignacio from Bogotá, Colombia. The protocol follows the Colombian national regulations and the Declaration of Helsinki. All the included subjects provided written informed consent.

## Author Contributions

SG-O recruited the donors, prepared the samples, designed and carried out the experiments, analyzed the data, made the figures, discussed the results, and wrote the paper. NB helped to prepare samples and perform experiments. ME wrote the first protocol of the study and standardized the flow cytometry settings. AR recruit IND patients. AM recruit heart failure patients (CCC and HTCC) and carried out their clinical assessment. CP and AC help with reagents, discussed the results, and reviewed the manuscript. JG acquired and administered funding resources, coordinated the study, designed the experiments, and wrote the manuscript. All authors contributed to manuscript revision and approved the submitted version.

### Conflict of Interest Statement

The authors declare that the research was conducted in the absence of any commercial or financial relationships that could be construed as a potential conflict of interest.

## Supplementary Material

Supplementary Figure 1 Gating strategy for 3-color flow cytometry with a sample from a representative CCC donor. Mononuclear cells were defined according to FSC vs. SSC parameters and gated on the singlets. HLA-DR+ population gate was determined based on the isotype control. On this population, it was defined the percentage of total monocytes (CD14+ CD16+ cells) and monocyte subsets according to CD14 and CD16 expression as follows: CD14++ CD16− (classical), CD14++ CD16+ (intermediate) and CD14+ CD16 ++ (non-classical).

Supplementary Figure 2 Mean fluorescence intensity (MFI) for HLA-DR expression in monocyte subsets from IND, CCC, HTCC, NCC and HI. Significant differences determined by Kruskal-Wallis test followed by Dunn's *post hoc* test are shown as follows: ^*^*p* < 0.05, ^**^*p* < 0.01, ^***^*p* < 0.001, ^****^*p* < 0.0001.

Supplementary Figure 3 Monocyte subsets and left ventricular ejection fraction (LVEF) variations associated with the time of heart transplantation and reactivation of *T. cruzi* infection. As shown for CCC16 and CCC18 patients, the percentages of classical monocytes increased and the percentage of intermediate monocytes diminished after heart transplantation, while IL-12p40 blood levels augmented in both patients. In the case of CCC21 patient, it was observed similar monocyte subsets changes upon antiparasitic treatment for parasite reactivation.
