# Supplementary figures and images for: Intermediate Monocytes and Cytokine Production Associated With Severe Forms of Chagas Disease
Source: Front Immunol. 2019 Jul 19;10:1671. doi: 10.3389/fimmu.2019.01671 (PMC6658923; doi:10.3389/fimmu.2019.01671)

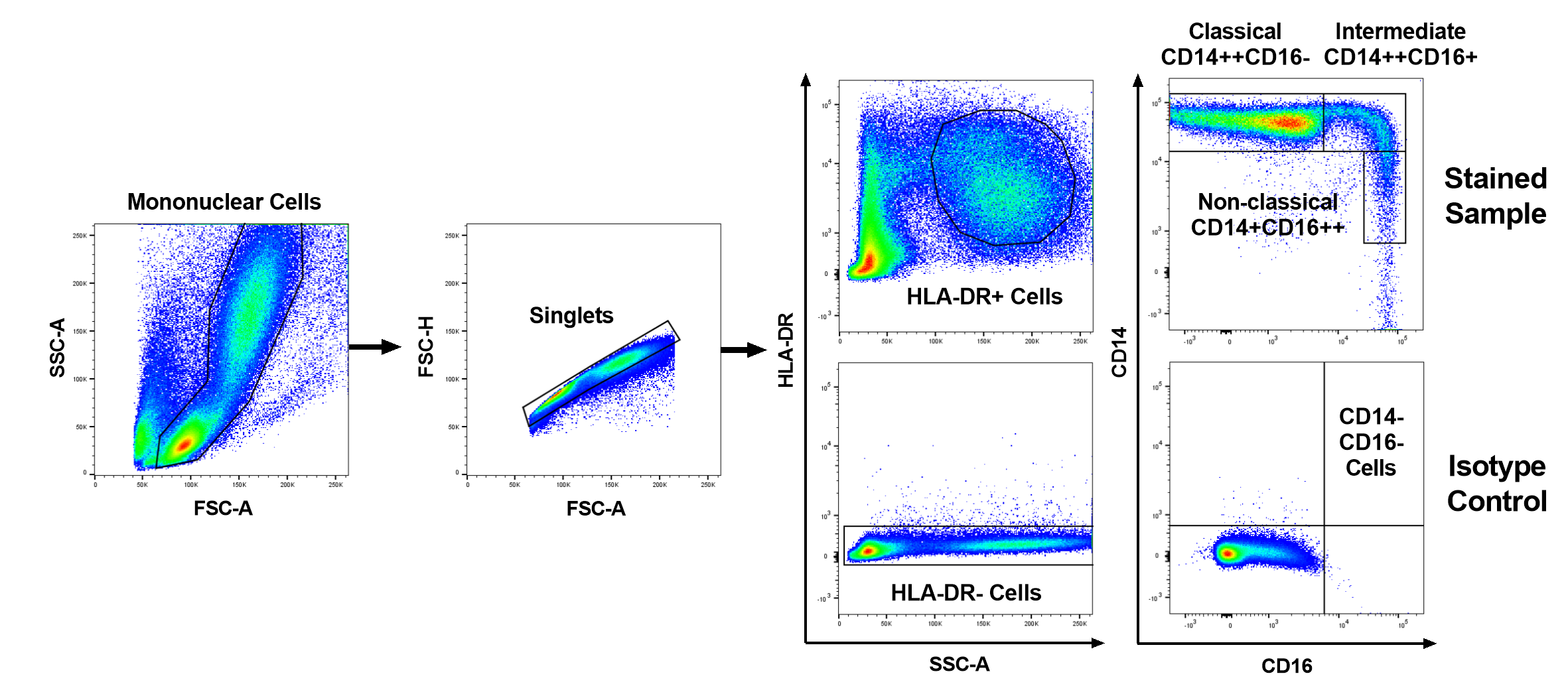

Supplement: Supplementary Figure 1 — Gating strategy for 3-color flow cytometry with a sample from a representative CCC donor. Mononuclear cells were defined according to FSC vs. SSC parameters and gated on the singlets. HLA-DR+ population gate was determined based on the isotype control. On this population, it was defined the percentage of total monocytes (CD14+ CD16+ cells) and monocyte subsets according to CD14 and CD16 expression as follows: CD14++ CD16− (classical), CD14++ CD16+ (intermediate) and CD14+ CD16 ++ (non-classical). [file Image_1.tif]

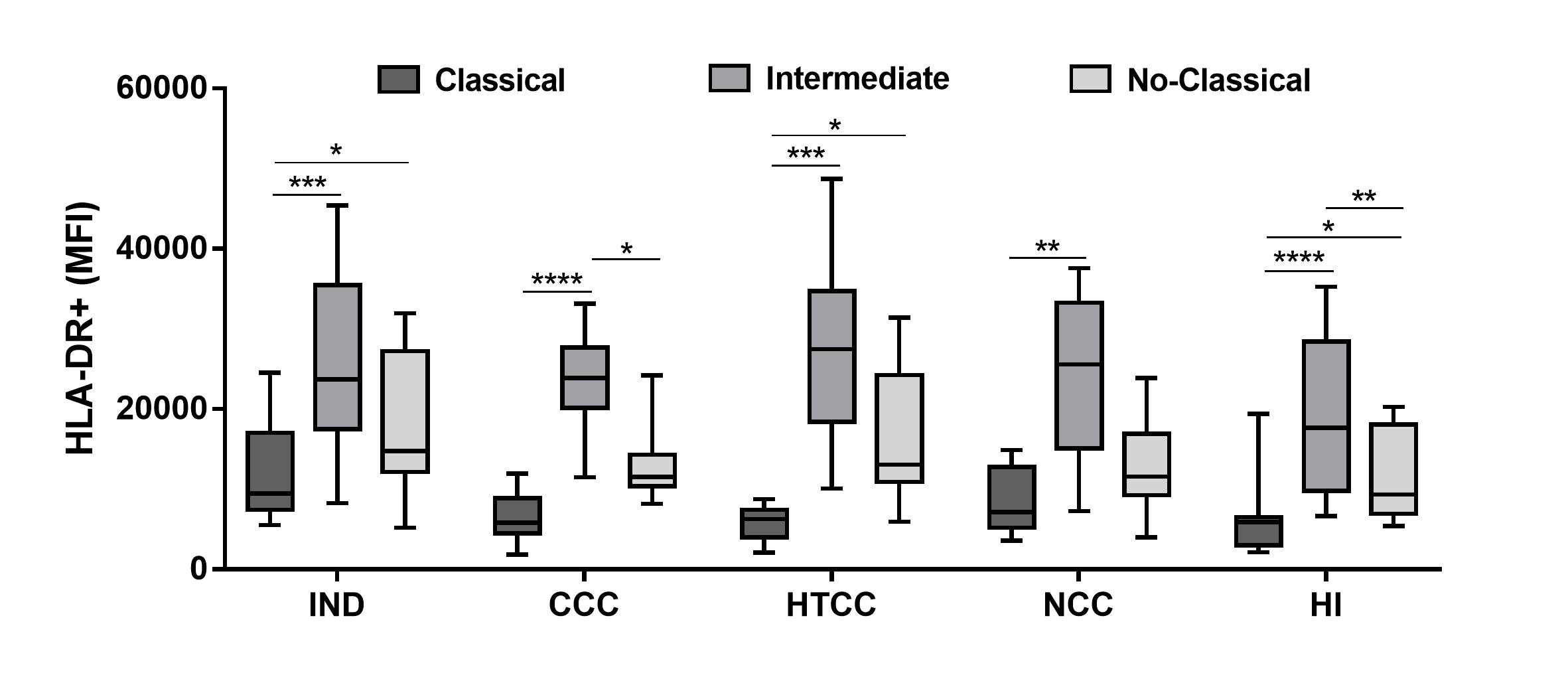

Supplement: Supplementary Figure 2 — Mean fluorescence intensity (MFI) for HLA-DR expression in monocyte subsets from IND, CCC, HTCC, NCC and HI. Significant differences determined by Kruskal-Wallis test followed by Dunn's post hoc test are shown as follows: *p < 0.05, **p < 0.01, ***p < 0.001, ****p < 0.0001. [file Image_2.TIF]

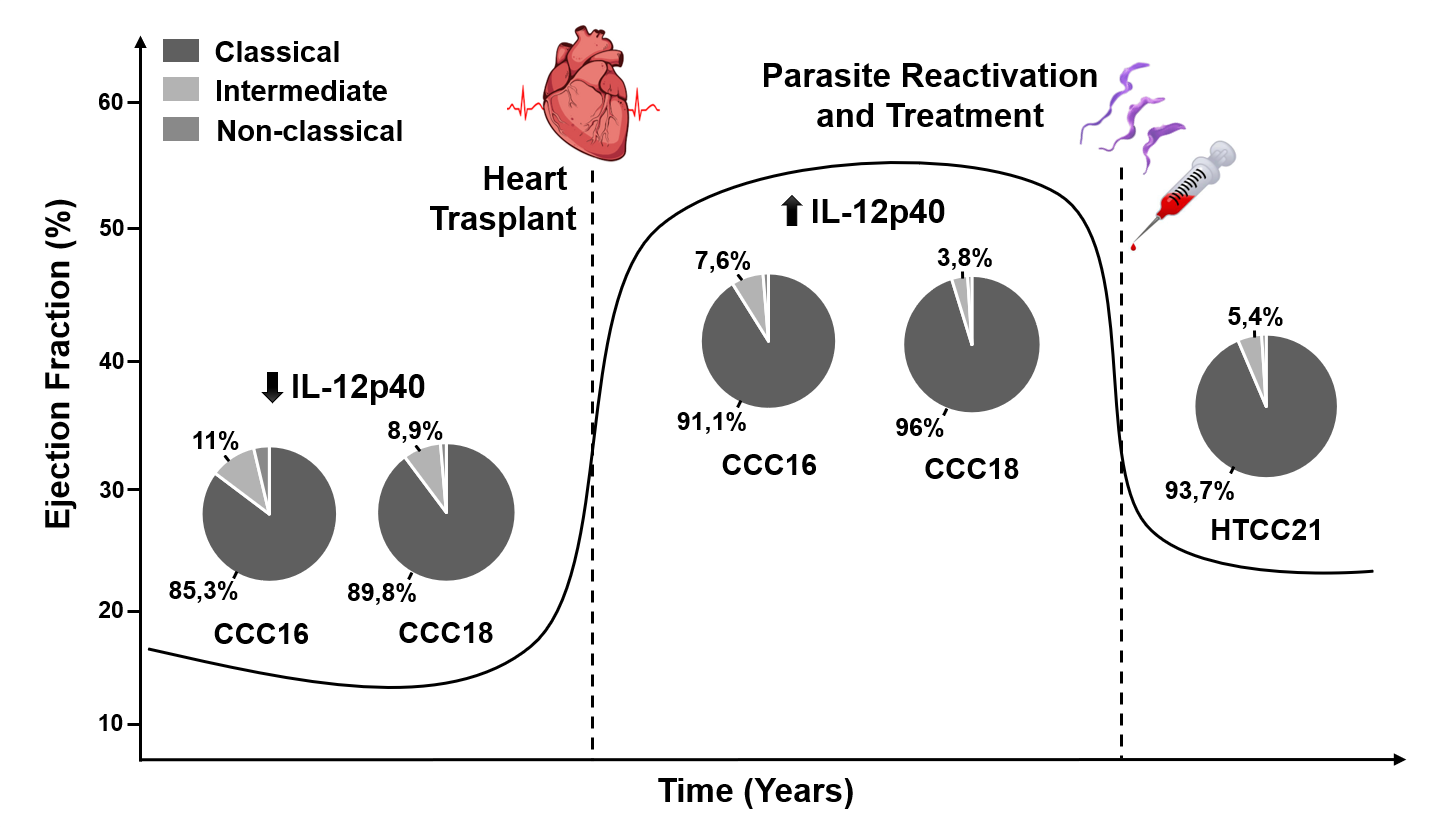

Supplement: Supplementary Figure 3 — Monocyte subsets and left ventricular ejection fraction (LVEF) variations associated with the time of heart transplantation and reactivation of T. cruzi infection. As shown for CCC16 and CCC18 patients, the percentages of classical monocytes increased and the percentage of intermediate monocytes diminished after heart transplantation, while IL-12p40 blood levels augmented in both patients. In the case of CCC21 patient, it was observed similar monocyte subsets changes upon antiparasitic treatment for parasite reactivation. [file Image_3.TIF]
